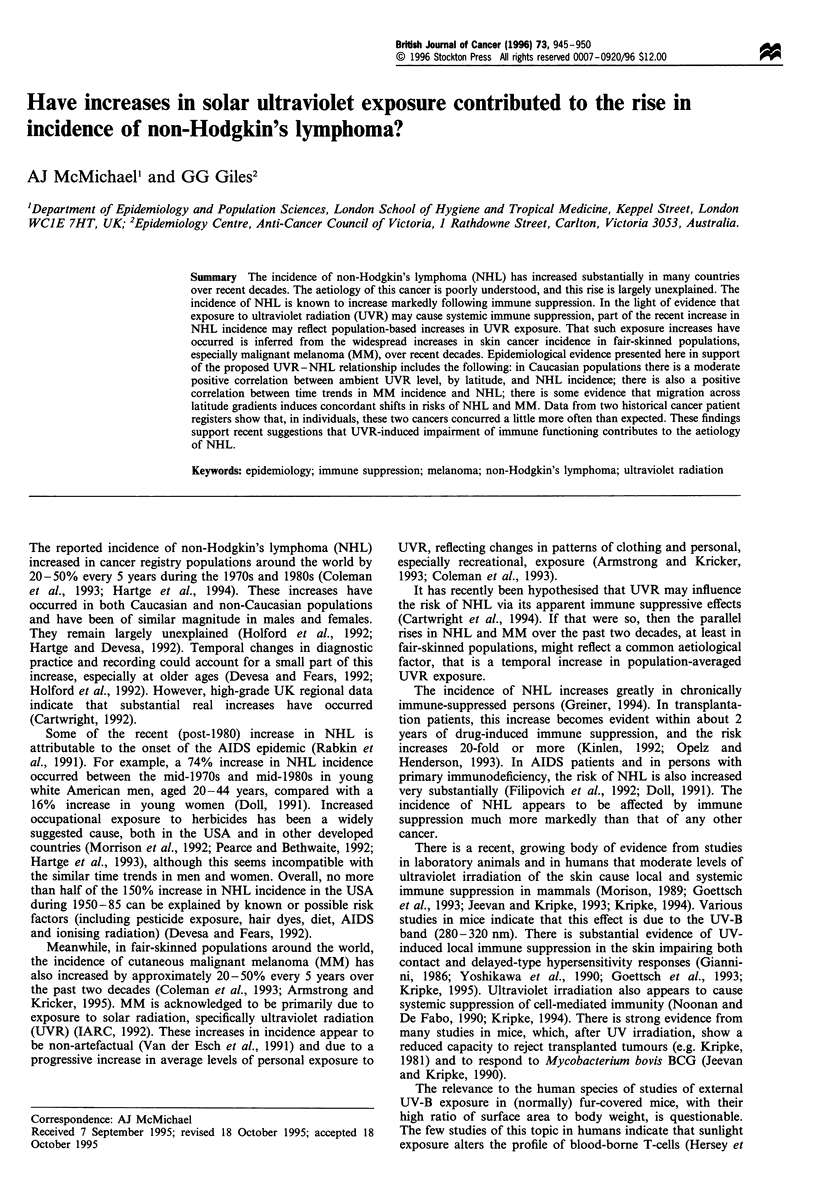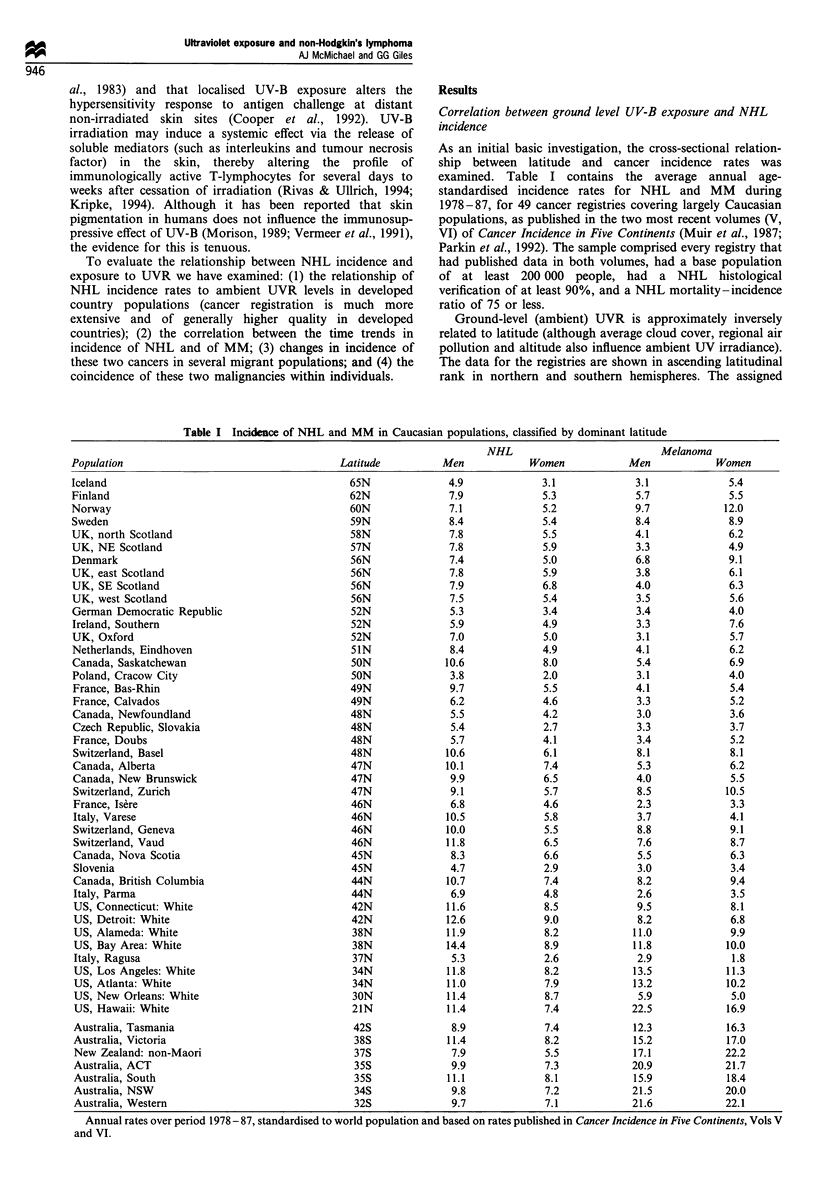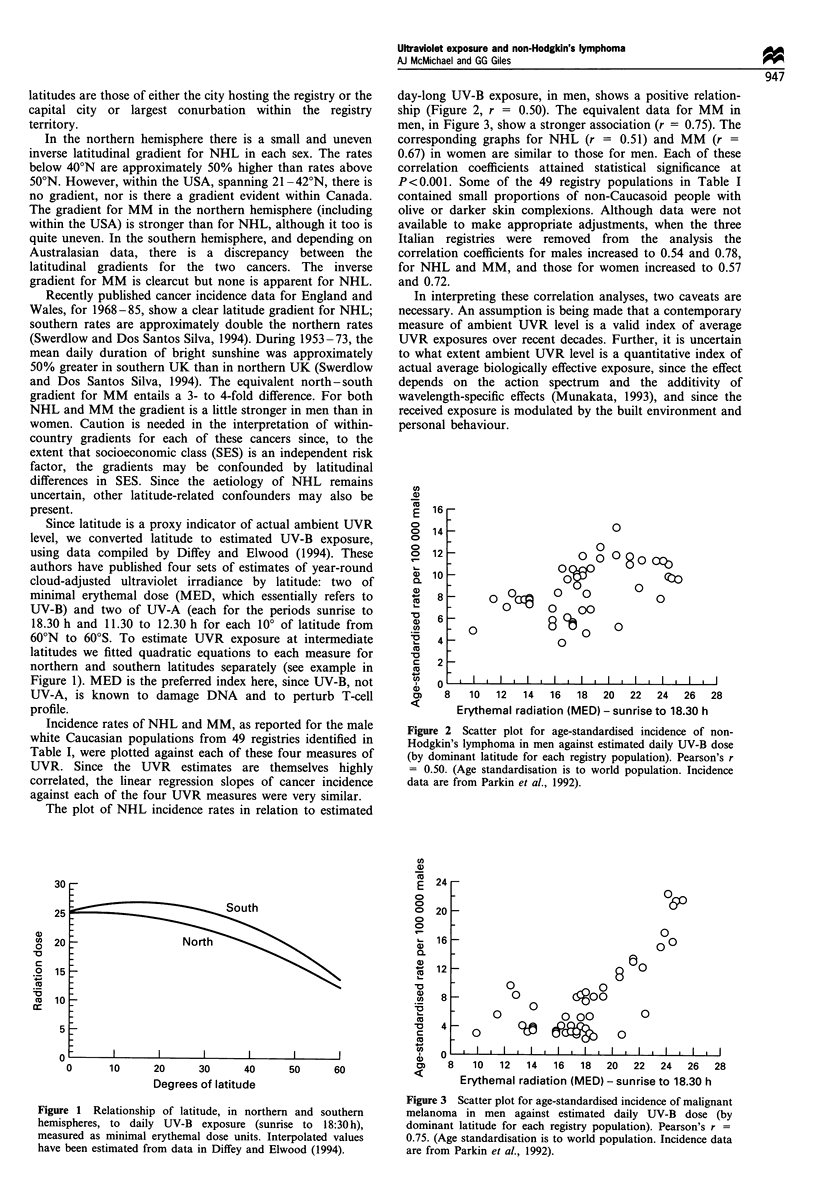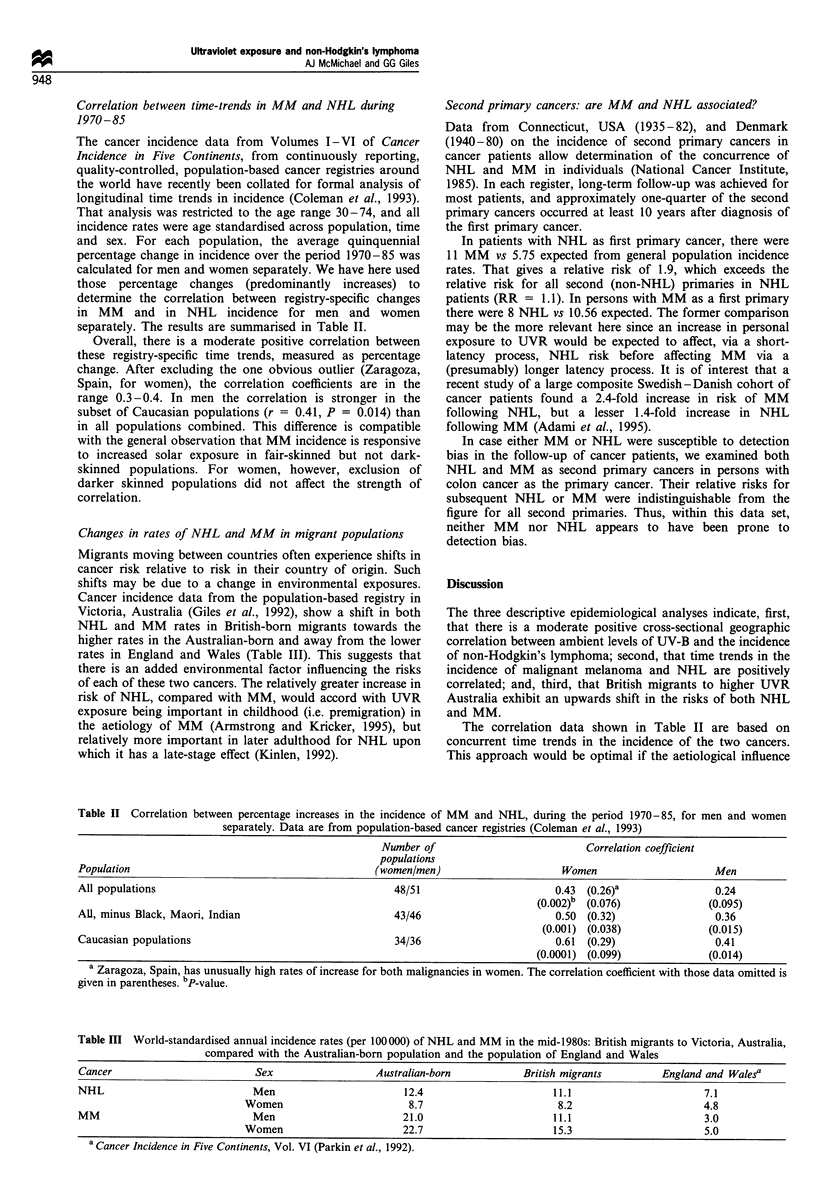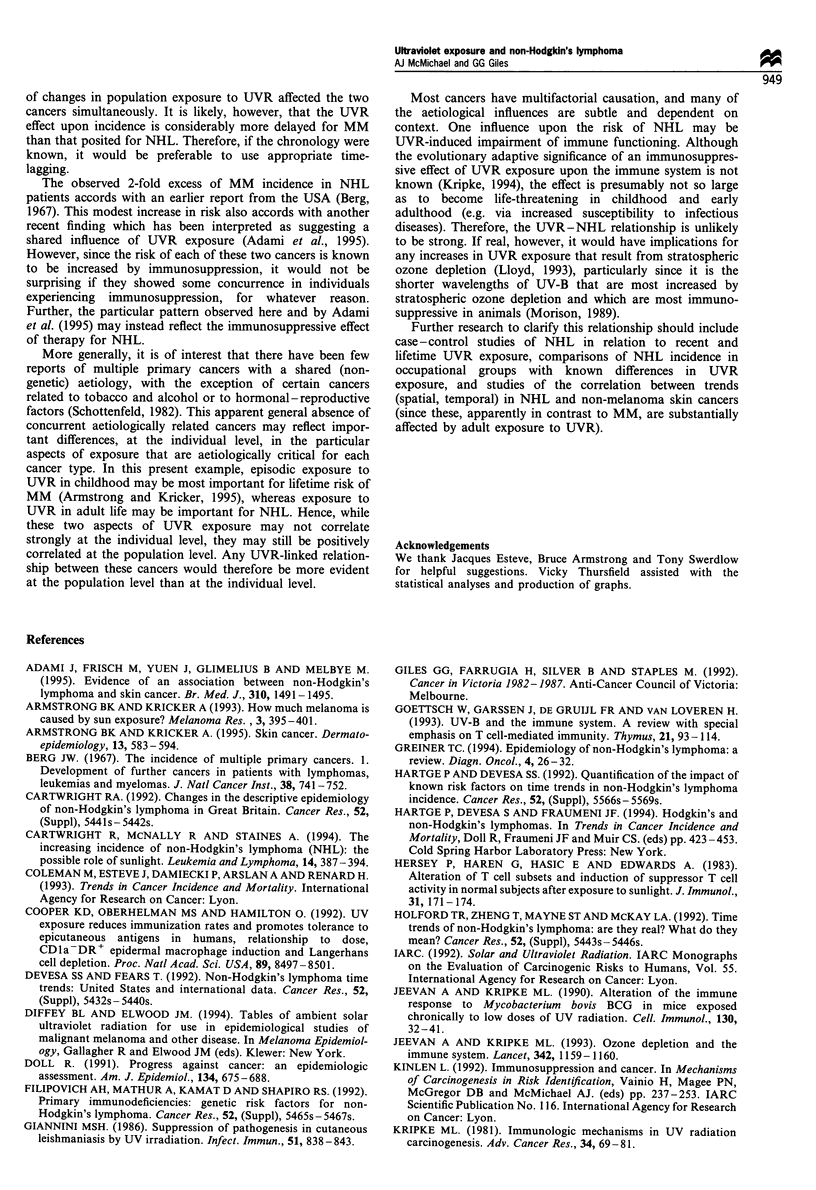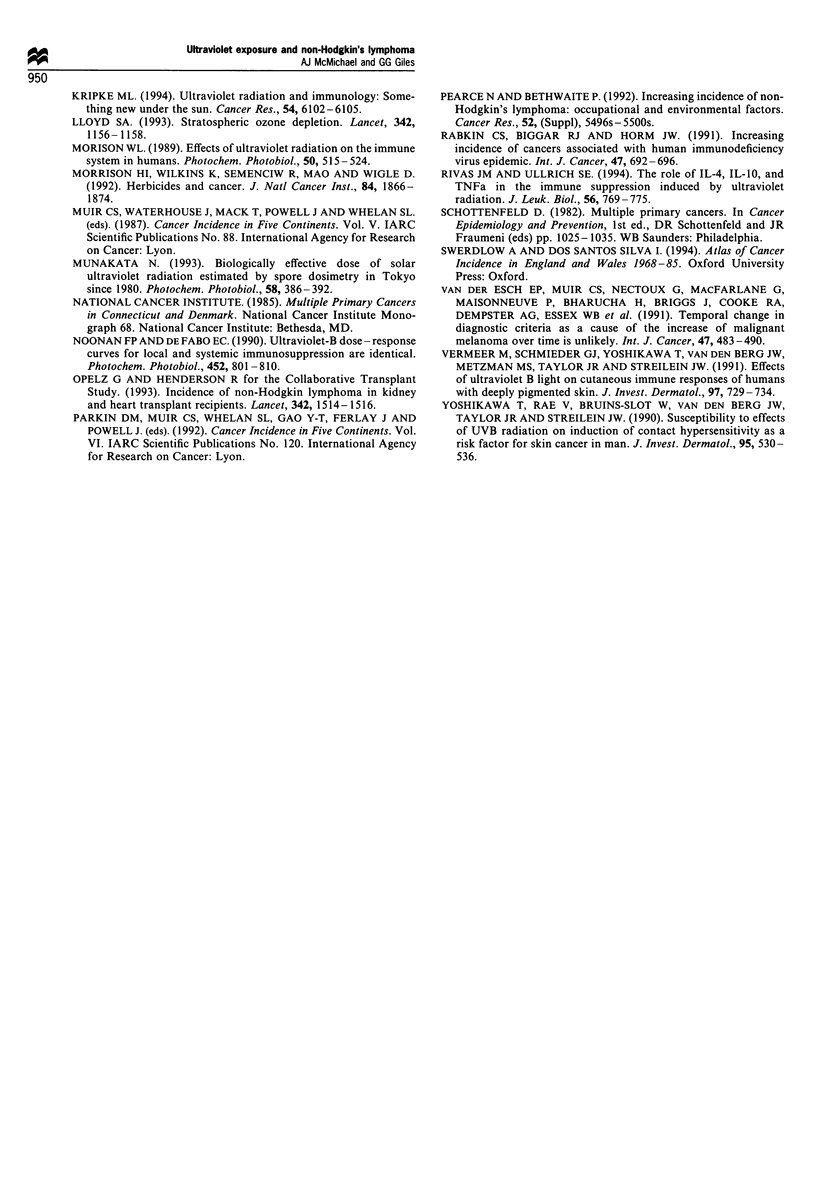# Have increases in solar ultraviolet exposure contributed to the rise in incidence of non-Hodgkin's lymphoma?

**DOI:** 10.1038/bjc.1996.169

**Published:** 1996-04

**Authors:** A. J. McMichael, G. G. Giles

**Affiliations:** Department of Epidemiology and Population Sciences, London School of Hygiene and Tropical Medicine, UK.

## Abstract

The incidence of non-Hodgkin's lymphoma (NHL) has increased substantially in many countries over recent decades. The aetiology of this cancer is poorly understood, and this rise is largely unexplained. The incidence of NHL is known to increase markedly following immune suppression. In the light of evidence that exposure to ultraviolet radiation (UVR) may cause systemic immune suppression, part of the recent increase in NHL incidence may reflect population-based increases in UVR exposure. That such exposure increases have occurred is inferred from the widespread increases in skin cancer incidence in fair-skinned populations, especially malignant melanoma (MM), over recent decades. Epidemiological evidence presented here in support of the proposed UVR-NHL relationship includes the following: in Caucasian populations there is a moderate positive correlation between ambient UVR level, by latitude, and NHL incidence; there is also a positive correlation between time trends in MM incidence and NHL; there is some evidence that migration across latitude gradients induces concordant shifts in risks of NHL and MM. Data from two historical cancer patient registers show that, in individuals, these two cancers concurred a little more often than expected. These findings support recent suggestions that UVR-induced impairment of immune functioning contributes to the aetiology of NHL.


British Journal of Cancer (1996) 73, 945-950
© 1996 Stockton Press All rights reserved 0007-0920/96 $12.00

### Have increases in solar ultraviolet exposure contributed to the rise in incidence of non-Hodgkin's lymphoma?

AJ McMichael[1] and GG Giles[2]

[1]*Department of Epidemiology and Population Sciences, London School of Hygiene and Tropical Medicine, Keppel Street, London WC1E 7HT, UK;* [2]*Epidemiology Centre, Anti-Cancer Council of Victoria, 1 Rathdowne Street, Carlton, Victoria 3053, Australia.*

**Summary** The incidence of non-Hodgkin's lymphoma (NHL) has increased substantially in many countries over recent decades. The aetiology of this cancer is poorly understood, and this rise is largely unexplained. The incidence of NHL is known to increase markedly following immune suppression. In the light of evidence that exposure to ultraviolet radiation (UVR) may cause systemic immune suppression, part of the recent increase in NHL incidence may reflect population-based increases in UVR exposure. That such exposure increases have occurred is inferred from the widespread increases in skin cancer incidence in fair-skinned populations, especially malignant melanoma (MM), over recent decades. Epidemiological evidence presented here in support of the proposed UVR-NHL relationship includes the following: in Caucasian populations there is a moderate positive correlation between ambient UVR level, by latitude, and NHL incidence; there is also a positive correlation between time trends in MM incidence and NHL; there is some evidence that migration across latitude gradients induces concordant shifts in risks of NHL and MM. Data from two historical cancer patient registers show that, in individuals, these two cancers concurred a little more often than expected. These findings support recent suggestions that UVR-induced impairment of immune functioning contributes to the aetiology of NHL.

**Keywords:** epidemiology; immune suppression; melanoma; non-Hodgkin's lymphoma; ultraviolet radiation

The reported incidence of non-Hodgkin's lymphoma (NHL) increased in cancer registry populations around the world by 20-50% every 5 years during the 1970s and 1980s (Coleman *et al.*, 1993; Hartge *et al.*, 1994). These increases have occurred in both Caucasian and non-Caucasian populations and have been of similar magnitude in males and females. They remain largely unexplained (Holford *et al.*, 1992; Hartge and Devesa, 1992). Temporal changes in diagnostic practice and recording could account for a small part of this increase, especially at older ages (Devesa and Fears, 1992; Holford *et al.*, 1992). However, high-grade UK regional data indicate that substantial real increases have occurred (Cartwright, 1992).

Some of the recent (post-1980) increase in NHL is attributable to the onset of the AIDS epidemic (Rabkin *et al.*, 1991). For example, a 74% increase in NHL incidence occurred between the mid-1970s and mid-1980s in young white American men, aged 20-44 years, compared with a 16% increase in young women (Doll, 1991). Increased occupational exposure to herbicides has been a widely suggested cause, both in the USA and in other developed countries (Morrison *et al.*, 1992; Pearce and Bethwaite, 1992; Hartge *et al.*, 1993), although this seems incompatible with the similar time trends in men and women. Overall, no more than half of the 150% increase in NHL incidence in the USA during 1950-85 can be explained by known or possible risk factors (including pesticide exposure, hair dyes, diet, AIDS and ionising radiation) (Devesa and Fears, 1992).

Meanwhile, in fair-skinned populations around the world, the incidence of cutaneous malignant melanoma (MM) has also increased by approximately 20-50% every 5 years over the past two decades (Coleman *et al.*, 1993; Armstrong and Kricker, 1995). MM is acknowledged to be primarily due to exposure to solar radiation, specifically ultraviolet radiation (UVR) (IARC, 1992). These increases in incidence appear to be non-artefactual (Van der Esch *et al.*, 1991) and due to a progressive increase in average levels of personal exposure to UVR, reflecting changes in patterns of clothing and personal, especially recreational, exposure (Armstrong and Kricker, 1993; Coleman *et al.*, 1993).

It has recently been hypothesised that UVR may influence the risk of NHL via its apparent immune suppressive effects (Cartwright *et al.*, 1994). If that were so, then the parallel rises in NHL and MM over the past two decades, at least in fair-skinned populations, might reflect a common aetiological factor, that is a temporal increase in population-averaged UVR exposure.

The incidence of NHL increases greatly in chronically immune-suppressed persons (Greiner, 1994). In transplantation patients, this increase becomes evident within about 2 years of drug-induced immune suppression, and the risk increases 20-fold or more (Kinlen, 1992; Opelz and Henderson, 1993). In AIDS patients and in persons with primary immunodeficiency, the risk of NHL is also increased very substantially (Filipovich *et al.*, 1992; Doll, 1991). The incidence of NHL appears to be affected by immune suppression much more markedly than that of any other cancer.

There is a recent, growing body of evidence from studies in laboratory animals and in humans that moderate levels of ultraviolet irradiation of the skin cause local and systemic immune suppression in mammals (Morison, 1989; Goettsch *et al.*, 1993; Jeevan and Kripke, 1993; Kripke, 1994). Various studies in mice indicate that this effect is due to the UV-B band (280-320 nm). There is substantial evidence of UV-induced local immune suppression in the skin impairing both contact and delayed-type hypersensitivity responses (Giannini, 1986; Yoshikawa *et al.*, 1990; Goettsch *et al.*, 1993; Kripke, 1995). Ultraviolet irradiation also appears to cause systemic suppression of cell-mediated immunity (Noonan and De Fabo, 1990; Kripke, 1994). There is strong evidence from many studies in mice, which, after UV irradiation, show a reduced capacity to reject transplanted tumours (e.g. Kripke, 1981) and to respond to *Mycobacterium bovis* BCG (Jeevan and Kripke, 1990).

The relevance to the human species of studies of external UV-B exposure in (normally) fur-covered mice, with their high ratio of surface area to body weight, is questionable. The few studies of this topic in humans indicate that sunlight exposure alters the profile of blood-borne T-cells (Hersey *et*

Correspondence: AJ McMichael
Received 7 September 1995; revised 18 October 1995; accepted 18 October 1995

*al.*, 1983) and that localised UV-B exposure alters the hypersensitivity response to antigen challenge at distant non-irradiated skin sites (Cooper *et al.*, 1992). UV-B irradiation may induce a systemic effect via the release of soluble mediators (such as interleukins and tumour necrosis factor) in the skin, thereby altering the profile of immunologically active T-lymphocytes for several days to weeks after cessation of irradiation (Rivas & Ullrich, 1994; Kripke, 1994). Although it has been reported that skin pigmentation in humans does not influence the immunosuppressive effect of UV-B (Morison, 1989; Vermeer *et al.*, 1991), the evidence for this is tenuous.

To evaluate the relationship between NHL incidence and exposure to UVR we have examined: (1) the relationship of NHL incidence rates to ambient UVR levels in developed country populations (cancer registration is much more extensive and of generally higher quality in developed countries); (2) the correlation between the time trends in incidence of NHL and of MM; (3) changes in incidence of these two cancers in several migrant populations; and (4) the coincidence of these two malignancies within individuals.

### Results

### *Correlation between ground level UV-B exposure and NHL incidence*

As an initial basic investigation, the cross-sectional relationship between latitude and cancer incidence rates was examined. Table I contains the average annual age-standardised incidence rates for NHL and MM during 1978–87, for 49 cancer registries covering largely Caucasian populations, as published in the two most recent volumes (V, VI) of *Cancer Incidence in Five Continents* (Muir *et al.*, 1987; Parkin *et al.*, 1992). The sample comprised every registry that had published data in both volumes, had a base population of at least 200 000 people, had a NHL histological verification of at least 90%, and a NHL mortality–incidence ratio of 75 or less.

Ground-level (ambient) UVR is approximately inversely related to latitude (although average cloud cover, regional air pollution and altitude also influence ambient UV irradiance). The data for the registries are shown in ascending latitudinal rank in northern and southern hemispheres. The assigned

**Table I** Incidence of NHL and MM in Caucasian populations, classified by dominant latitude

| *Population* | *Latitude* | *NHL* *Men* | *NHL* *Women* | *Melanoma* *Men* | *Melanoma* *Women* |
|---|---|---|---|---|---|
| Iceland | 65N | 4.9 | 3.1 | 3.1 | 5.4 |
| Finland | 62N | 7.9 | 5.3 | 5.7 | 5.5 |
| Norway | 60N | 7.1 | 5.2 | 9.7 | 12.0 |
| Sweden | 59N | 8.4 | 5.4 | 8.4 | 8.9 |
| UK, north Scotland | 58N | 7.8 | 5.5 | 4.1 | 6.2 |
| UK, NE Scotland | 57N | 7.8 | 5.9 | 3.3 | 4.9 |
| Denmark | 56N | 7.4 | 5.0 | 6.8 | 9.1 |
| UK, east Scotland | 56N | 7.8 | 5.9 | 3.8 | 6.1 |
| UK, SE Scotland | 56N | 7.9 | 6.8 | 4.0 | 6.3 |
| UK, west Scotland | 56N | 7.5 | 5.4 | 3.5 | 5.6 |
| German Democratic Republic | 52N | 5.3 | 3.4 | 3.4 | 4.0 |
| Ireland, Southern | 52N | 5.9 | 4.9 | 3.3 | 7.6 |
| UK, Oxford | 52N | 7.0 | 5.0 | 3.1 | 5.7 |
| Netherlands, Eindhoven | 51N | 8.4 | 4.9 | 4.1 | 6.2 |
| Canada, Saskatchewan | 50N | 10.6 | 8.0 | 5.4 | 6.9 |
| Poland, Cracow City | 50N | 3.8 | 2.0 | 3.1 | 4.0 |
| France, Bas-Rhin | 49N | 9.7 | 5.5 | 4.1 | 5.4 |
| France, Calvados | 49N | 6.2 | 4.6 | 3.3 | 5.2 |
| Canada, Newfoundland | 48N | 5.5 | 4.2 | 3.0 | 3.6 |
| Czech Republic, Slovakia | 48N | 5.4 | 2.7 | 3.3 | 3.7 |
| France, Doubs | 48N | 5.7 | 4.1 | 3.4 | 5.2 |
| Switzerland, Basel | 48N | 10.6 | 6.1 | 8.1 | 8.1 |
| Canada, Alberta | 47N | 10.1 | 7.4 | 5.3 | 6.2 |
| Canada, New Brunswick | 47N | 9.9 | 6.5 | 4.0 | 5.5 |
| Switzerland, Zurich | 47N | 9.1 | 5.7 | 8.5 | 10.5 |
| France, Isère | 46N | 6.8 | 4.6 | 2.3 | 3.3 |
| Italy, Varese | 46N | 10.5 | 5.8 | 3.7 | 4.1 |
| Switzerland, Geneva | 46N | 10.0 | 5.5 | 8.8 | 9.1 |
| Switzerland, Vaud | 46N | 11.8 | 6.5 | 7.6 | 8.7 |
| Canada, Nova Scotia | 45N | 8.3 | 6.6 | 5.5 | 6.3 |
| Slovenia | 45N | 4.7 | 2.9 | 3.0 | 3.4 |
| Canada, British Columbia | 44N | 10.7 | 7.4 | 8.2 | 9.4 |
| Italy, Parma | 44N | 6.9 | 4.8 | 2.6 | 3.5 |
| US, Connecticut: White | 42N | 11.6 | 8.5 | 9.5 | 8.1 |
| US, Detroit: White | 42N | 12.6 | 9.0 | 8.2 | 6.8 |
| US, Alameda: White | 38N | 11.9 | 8.2 | 11.0 | 9.9 |
| US, Bay Area: White | 38N | 14.4 | 8.9 | 11.8 | 10.0 |
| Italy, Ragusa | 37N | 5.3 | 2.6 | 2.9 | 1.8 |
| US, Los Angeles: White | 34N | 11.8 | 8.2 | 13.5 | 11.3 |
| US, Atlanta: White | 34N | 11.0 | 7.9 | 13.2 | 10.2 |
| US, New Orleans: White | 30N | 11.4 | 8.7 | 5.9 | 5.0 |
| US, Hawaii: White | 21N | 11.4 | 7.4 | 22.5 | 16.9 |
| Australia, Tasmania | 42S | 8.9 | 7.4 | 12.3 | 16.3 |
| Australia, Victoria | 38S | 11.4 | 8.2 | 15.2 | 17.0 |
| New Zealand: non-Maori | 37S | 7.9 | 5.5 | 17.1 | 22.2 |
| Australia, ACT | 35S | 9.9 | 7.3 | 20.9 | 21.7 |
| Australia, South | 35S | 11.1 | 8.1 | 15.9 | 18.4 |
| Australia, NSW | 34S | 9.8 | 7.2 | 21.5 | 20.0 |
| Australia, Western | 32S | 9.7 | 7.1 | 21.6 | 22.1 |

Annual rates over period 1978–87, standardised to world population and based on rates published in *Cancer Incidence in Five Continents*, Vols V and VI.

latitudes are those of either the city hosting the registry or the capital city or largest conurbation within the registry territory.

In the northern hemisphere there is a small and uneven inverse latitudinal gradient for NHL in each sex. The rates below 40°N are approximately 50% higher than rates above 50°N. However, within the USA, spanning 21–42°N, there is no gradient, nor is there a gradient evident within Canada. The gradient for MM in the northern hemisphere (including within the USA) is stronger than for NHL, although it too is quite uneven. In the southern hemisphere, and depending on Australasian data, there is a discrepancy between the latitudinal gradients for the two cancers. The inverse gradient for MM is clearcut but none is apparent for NHL.

Recently published cancer incidence data for England and Wales, for 1968–85, show a clear latitude gradient for NHL; southern rates are approximately double the northern rates (Swerdlow and Dos Santos Silva, 1994). During 1953–73, the mean daily duration of bright sunshine was approximately 50% greater in southern UK than in northern UK (Swerdlow and Dos Santos Silva, 1994). The equivalent north–south gradient for MM entails a 3- to 4-fold difference. For both NHL and MM the gradient is a little stronger in men than in women. Caution is needed in the interpretation of within-country gradients for each of these cancers since, to the extent that socioeconomic class (SES) is an independent risk factor, the gradients may be confounded by latitudinal differences in SES. Since the aetiology of NHL remains uncertain, other latitude-related confounders may also be present.

Since latitude is a proxy indicator of actual ambient UVR level, we converted latitude to estimated UV-B exposure, using data compiled by Diffey and Elwood (1994). These authors have published four sets of estimates of year-round cloud-adjusted ultraviolet irradiance by latitude: two of minimal erythemal dose (MED, which essentially refers to UV-B) and two of UV-A (each for the periods sunrise to 18.30 h and 11.30 to 12.30 h for each 10° of latitude from 60°N to 60°S. To estimate UVR exposure at intermediate latitudes we fitted quadratic equations to each measure for northern and southern latitudes separately (see example in Figure 1). MED is the preferred index here, since UV-B, not UV-A, is known to damage DNA and to perturb T-cell profile.

Incidence rates of NHL and MM, as reported for the male white Caucasian populations from 49 registries identified in Table I, were plotted against each of these four measures of UVR. Since the UVR estimates are themselves highly correlated, the linear regression slopes of cancer incidence against each of the four UVR measures were very similar.

The plot of NHL incidence rates in relation to estimated day-long UV-B exposure, in men, shows a positive relationship (Figure 2, $r$ = 0.50). The equivalent data for MM in men, in Figure 3, show a stronger association ($r$ = 0.75). The corresponding graphs for NHL ($r$ = 0.51) and MM ($r$ = 0.67) in women are similar to those for men. Each of these correlation coefficients attained statistical significance at $P<0.001$. Some of the 49 registry populations in Table I contained small proportions of non-Caucasoid people with olive or darker skin complexions. Although data were not available to make appropriate adjustments, when the three Italian registries were removed from the analysis the correlation coefficients for males increased to 0.54 and 0.78, for NHL and MM, and those for women increased to 0.57 and 0.72.

In interpreting these correlation analyses, two caveats are necessary. An assumption is being made that a contemporary measure of ambient UVR level is a valid index of average UVR exposures over recent decades. Further, it is uncertain to what extent ambient UVR level is a quantitative index of actual average biologically effective exposure, since the effect depends on the action spectrum and the additivity of wavelength-specific effects (Munakata, 1993), and since the received exposure is modulated by the built environment and personal behaviour.

**Figure 2** Scatter plot for age-standardised incidence of non-Hodgkin's lymphoma in men against estimated daily UV-B dose (by dominant latitude for each registry population). Pearson's $r$ = 0.50. (Age standardisation is to world population. Incidence data are from Parkin *et al.*, 1992).

**Figure 1** Relationship of latitude, in northern and southern hemispheres, to daily UV-B exposure (sunrise to 18:30 h), measured as minimal erythemal dose units. Interpolated values have been estimated from data in Diffey and Elwood (1994).

**Figure 3** Scatter plot for age-standardised incidence of malignant melanoma in men against estimated daily UV-B dose (by dominant latitude for each registry population). Pearson's $r$ = 0.75. (Age standardisation is to world population. Incidence data are from Parkin *et al.*, 1992).

*Correlation between time-trends in MM and NHL during 1970–85*

The cancer incidence data from Volumes I–VI of *Cancer Incidence in Five Continents*, from continuously reporting, quality-controlled, population-based cancer registries around the world have recently been collated for formal analysis of longitudinal time trends in incidence (Coleman *et al.*, 1993). That analysis was restricted to the age range 30–74, and all incidence rates were age standardised across population, time and sex. For each population, the average quinquennial percentage change in incidence over the period 1970–85 was calculated for men and women separately. We have here used those percentage changes (predominantly increases) to determine the correlation between registry-specific changes in MM and in NHL incidence for men and women separately. The results are summarised in Table II.

Overall, there is a moderate positive correlation between these registry-specific time trends, measured as percentage change. After excluding the one obvious outlier (Zaragoza, Spain, for women), the correlation coefficients are in the range 0.3–0.4. In men the correlation is stronger in the subset of Caucasian populations ($r = 0.41$, $P = 0.014$) than in all populations combined. This difference is compatible with the general observation that MM incidence is responsive to increased solar exposure in fair-skinned but not dark-skinned populations. For women, however, exclusion of darker skinned populations did not affect the strength of correlation.

*Changes in rates of NHL and MM in migrant populations*

Migrants moving between countries often experience shifts in cancer risk relative to risk in their country of origin. Such shifts may be due to a change in environmental exposures. Cancer incidence data from the population-based registry in Victoria, Australia (Giles *et al.*, 1992), show a shift in both NHL and MM rates in British-born migrants towards the higher rates in the Australian-born and away from the lower rates in England and Wales (Table III). This suggests that there is an added environmental factor influencing the risks of each of these two cancers. The relatively greater increase in risk of NHL, compared with MM, would accord with UVR exposure being important in childhood (i.e. premigration) in the aetiology of MM (Armstrong and Kricker, 1995), but relatively more important in later adulthood for NHL upon which it has a late-stage effect (Kinlen, 1992).

*Second primary cancers: are MM and NHL associated?*

Data from Connecticut, USA (1935–82), and Denmark (1940–80) on the incidence of second primary cancers in cancer patients allow determination of the concurrence of NHL and MM in individuals (National Cancer Institute, 1985). In each register, long-term follow-up was achieved for most patients, and approximately one-quarter of the second primary cancers occurred at least 10 years after diagnosis of the first primary cancer.

In patients with NHL as first primary cancer, there were 11 MM *vs* 5.75 expected from general population incidence rates. That gives a relative risk of 1.9, which exceeds the relative risk for all second (non-NHL) primaries in NHL patients (RR = 1.1). In persons with MM as a first primary there were 8 NHL *vs* 10.56 expected. The former comparison may be the more relevant here since an increase in personal exposure to UVR would be expected to affect, via a short-latency process, NHL risk before affecting MM via a (presumably) longer latency process. It is of interest that a recent study of a large composite Swedish–Danish cohort of cancer patients found a 2.4-fold increase in risk of MM following NHL, but a lesser 1.4-fold increase in NHL following MM (Adami *et al.*, 1995).

In case either MM or NHL were susceptible to detection bias in the follow-up of cancer patients, we examined both NHL and MM as second primary cancers in persons with colon cancer as the primary cancer. Their relative risks for subsequent NHL or MM were indistinguishable from the figure for all second primaries. Thus, within this data set, neither MM nor NHL appears to have been prone to detection bias.

### Discussion

The three descriptive epidemiological analyses indicate, first, that there is a moderate positive cross-sectional geographic correlation between ambient levels of UV-B and the incidence of non-Hodgkin's lymphoma; second, that time trends in the incidence of malignant melanoma and NHL are positively correlated; and, third, that British migrants to higher UVR Australia exhibit an upwards shift in the risks of both NHL and MM.

The correlation data shown in Table II are based on concurrent time trends in the incidence of the two cancers. This approach would be optimal if the aetiological influence

**Table II** Correlation between percentage increases in the incidence of MM and NHL, during the period 1970–85, for men and women separately. Data are from population-based cancer registries (Coleman *et al.*, 1993)

| *Population* | *Number of populations (women/men)* | *Correlation coefficient* | | |
|---|---|---|---|---|
| | | *Women* | | *Men* |
| All populations | 48/51 | 0.43 | (0.26)[a] | 0.24 |
| | | (0.002)[b] | (0.076) | (0.095) |
| All, minus Black, Maori, Indian | 43/46 | 0.50 | (0.32) | 0.36 |
| | | (0.001) | (0.038) | (0.015) |
| Caucasian populations | 34/36 | 0.61 | (0.29) | 0.41 |
| | | (0.0001) | (0.099) | (0.014) |

[a] Zaragoza, Spain, has unusually high rates of increase for both malignancies in women. The correlation coefficient with those data omitted is given in parentheses. [b] *P*-value.

**Table III** World-standardised annual incidence rates (per 100 000) of NHL and MM in the mid-1980s: British migrants to Victoria, Australia, compared with the Australian-born population and the population of England and Wales

| *Cancer* | *Sex* | *Australian-born* | *British migrants* | *England and Wales*[a] |
|---|---|---|---|---|
| NHL | Men | 12.4 | 11.1 | 7.1 |
| | Women | 8.7 | 8.2 | 4.8 |
| MM | Men | 21.0 | 11.1 | 3.0 |
| | Women | 22.7 | 15.3 | 5.0 |

[a] *Cancer Incidence in Five Continents*, Vol. VI (Parkin *et al.*, 1992).

of changes in population exposure to UVR affected the two cancers simultaneously. It is likely, however, that the UVR effect upon incidence is considerably more delayed for MM than that posited for NHL. Therefore, if the chronology were known, it would be preferable to use appropriate time-lagging.

The observed 2-fold excess of MM incidence in NHL patients accords with an earlier report from the USA (Berg, 1967). This modest increase in risk also accords with another recent finding which has been interpreted as suggesting a shared influence of UVR exposure (Adami *et al.*, 1995). However, since the risk of each of these two cancers is known to be increased by immunosuppression, it would not be surprising if they showed some concurrence in individuals experiencing immunosuppression, for whatever reason. Further, the particular pattern observed here and by Adami *et al.* (1995) may instead reflect the immunosuppressive effect of therapy for NHL.

More generally, it is of interest that there have been few reports of multiple primary cancers with a shared (non-genetic) aetiology, with the exception of certain cancers related to tobacco and alcohol or to hormonal–reproductive factors (Schottenfeld, 1982). This apparent general absence of concurrent aetiologically related cancers may reflect important differences, at the individual level, in the particular aspects of exposure that are aetiologically critical for each cancer type. In this present example, episodic exposure to UVR in childhood may be most important for lifetime risk of MM (Armstrong and Kricker, 1995), whereas exposure to UVR in adult life may be important for NHL. Hence, while these two aspects of UVR exposure may not correlate strongly at the individual level, they may still be positively correlated at the population level. Any UVR-linked relationship between these cancers would therefore be more evident at the population level than at the individual level.

Most cancers have multifactorial causation, and many of the aetiological influences are subtle and dependent on context. One influence upon the risk of NHL may be UVR-induced impairment of immune functioning. Although the evolutionary adaptive significance of an immunosuppressive effect of UVR exposure upon the immune system is not known (Kripke, 1994), the effect is presumably not so large as to become life-threatening in childhood and early adulthood (e.g. via increased susceptibility to infectious diseases). Therefore, the UVR–NHL relationship is unlikely to be strong. If real, however, it would have implications for any increases in UVR exposure that result from stratospheric ozone depletion (Lloyd, 1993), particularly since it is the shorter wavelengths of UV-B that are most increased by stratospheric ozone depletion and which are most immunosuppressive in animals (Morison, 1989).

Further research to clarify this relationship should include case–control studies of NHL in relation to recent and lifetime UVR exposure, comparisons of NHL incidence in occupational groups with known differences in UVR exposure, and studies of the correlation between trends (spatial, temporal) in NHL and non-melanoma skin cancers (since these, apparently in contrast to MM, are substantially affected by adult exposure to UVR).

**Acknowledgements**
We thank Jacques Esteve, Bruce Armstrong and Tony Swerdlow for helpful suggestions. Vicky Thursfield assisted with the statistical analyses and production of graphs.

### References

ADAMI J, FRISCH M, YUEN J, GLIMELIUS B AND MELBYE M. (1995). Evidence of an association between non-Hodgkin's lymphoma and skin cancer. *Br. Med. J.*, **310**, 1491–1495.

ARMSTRONG BK AND KRICKER A (1993). How much melanoma is caused by sun exposure? *Melanoma Res.* , **3**, 395–401.

ARMSTRONG BK AND KRICKER A. (1995). Skin cancer. *Dermato-epidemiology*, **13**, 583–594.

BERG JW. (1967). The incidence of multiple primary cancers. 1. Development of further cancers in patients with lymphomas, leukemias and myelomas. *J. Natl Cancer Inst.*, **38**, 741–752.

CARTWRIGHT RA. (1992). Changes in the descriptive epidemiology of non-Hodgkin's lymphoma in Great Britain. *Cancer Res.*, **52**, (Suppl), 5441s–5442s.

CARTWRIGHT R, MCNALLY R AND STAINES A. (1994). The increasing incidence of non-Hodgkin's lymphoma (NHL): the possible role of sunlight. *Leukemia and Lymphoma*, **14**, 387–394.

COLEMAN M, ESTEVE J, DAMIECKI P, ARSLAN A AND RENARD H. (1993). *Trends in Cancer Incidence and Mortality*. International Agency for Research on Cancer: Lyon.

COOPER KD, OBERHELMAN MS AND HAMILTON O. (1992). UV exposure reduces immunization rates and promotes tolerance to epicutaneous antigens in humans, relationship to dose, $CD1a^-DR^+$ epidermal macrophage induction and Langerhans cell depletion. *Proc. Natl Acad. Sci. USA*, **89**, 8497–8501.

DEVESA SS AND FEARS T. (1992). Non-Hodgkin's lymphoma time trends: United States and international data. *Cancer Res.*, **52**, (Suppl), 5432s–5440s.

DIFFEY BL AND ELWOOD JM. (1994). Tables of ambient solar ultraviolet radiation for use in epidemiological studies of malignant melanoma and other disease. In *Melanoma Epidemiology*, Gallagher R and Elwood JM (eds). Klewer: New York.

DOLL R. (1991). Progress against cancer: an epidemiologic assessment. *Am. J. Epidemiol.*, **134**, 675–688.

FILIPOVICH AH, MATHUR A, KAMAT D AND SHAPIRO RS. (1992). Primary immunodeficiencies: genetic risk factors for non-Hodgkin's lymphoma. *Cancer Res.*, **52**, (Suppl), 5465s–5467s.

GIANNINI MSH. (1986). Suppression of pathogenesis in cutaneous leishmaniasis by UV irradiation. *Infect. Immun.*, **51**, 838–843.

GILES GG, FARRUGIA H, SILVER B AND STAPLES M. (1992). *Cancer in Victoria 1982–1987*. Anti-Cancer Council of Victoria: Melbourne.

GOETTSCH W, GARSSEN J, DE GRUIJL FR AND VAN LOVEREN H. (1993). UV-B and the immune system. A review with special emphasis on T cell-mediated immunity. *Thymus*, **21**, 93–114.

GREINER TC. (1994). Epidemiology of non-Hodgkin's lymphoma: a review. *Diagn. Oncol.*, **4**, 26–32.

HARTGE P AND DEVESA SS. (1992). Quantification of the impact of known risk factors on time trends in non-Hodgkin's lymphoma incidence. *Cancer Res.*, **52**, (Suppl), 5566s–5569s.

HARTGE P, DEVESA S AND FRAUMENI JF. (1994). Hodgkin's and non-Hodgkin's lymphomas. In *Trends in Cancer Incidence and Mortality*, Doll R, Fraumeni JF and Muir CS. (eds) pp. 423–453. Cold Spring Harbor Laboratory Press: New York.

HERSEY P, HAREN G, HASIC E AND EDWARDS A. (1983). Alteration of T cell subsets and induction of suppressor T cell activity in normal subjects after exposure to sunlight. *J. Immunol.*, **31**, 171–174.

HOLFORD TR, ZHENG T, MAYNE ST AND MCKAY LA. (1992). Time trends of non-Hodgkin's lymphoma: are they real? What do they mean? *Cancer Res.*, **52**, (Suppl), 5443s–5446s.

IARC. (1992). *Solar and Ultraviolet Radiation*. IARC Monographs on the Evaluation of Carcinogenic Risks to Humans, Vol. 55. International Agency for Research on Cancer: Lyon.

JEEVAN A AND KRIPKE ML. (1990). Alteration of the immune response to *Mycobacterium bovis* BCG in mice exposed chronically to low doses of UV radiation. *Cell. Immunol.*, **130**, 32–41.

JEEVAN A AND KRIPKE ML. (1993). Ozone depletion and the immune system. *Lancet*, **342**, 1159–1160.

KINLEN L. (1992). Immunosuppression and cancer. In *Mechanisms of Carcinogenesis in Risk Identification*, Vainio H, Magee PN, McGregor DB and McMichael AJ. (eds) pp. 237–253. IARC Scientific Publication No. 116. International Agency for Research on Cancer: Lyon.

KRIPKE ML. (1981). Immunologic mechanisms in UV radiation carcinogenesis. *Adv. Cancer Res.*, **34**, 69–81.

KRIPKE ML. (1994). Ultraviolet radiation and immunology: Something new under the sun. *Cancer Res.*, **54,** 6102–6105.

LLOYD SA. (1993). Stratospheric ozone depletion. *Lancet*, **342,** 1156–1158.

MORISON WL. (1989). Effects of ultraviolet radiation on the immune system in humans. *Photochem. Photobiol.*, **50,** 515–524.

MORRISON HI, WILKINS K, SEMENCIW R, MAO AND WIGLE D. (1992). Herbicides and cancer. *J. Natl Cancer Inst.*, **84,** 1866–1874.

MUIR CS, WATERHOUSE J, MACK T, POWELL J AND WHELAN SL. (eds). (1987). *Cancer Incidence in Five Continents.* Vol. V. IARC Scientific Publications No. 88. International Agency for Research on Cancer: Lyon.

MUNAKATA N. (1993). Biologically effective dose of solar ultraviolet radiation estimated by spore dosimetry in Tokyo since 1980. *Photochem. Photobiol.*, **58,** 386–392.

NATIONAL CANCER INSTITUTE. (1985). *Multiple Primary Cancers in Connecticut and Denmark.* National Cancer Institute Monograph 68. National Cancer Institute: Bethesda, MD.

NOONAN FP AND DE FABO EC. (1990). Ultraviolet-B dose–response curves for local and systemic immunosuppression are identical. *Photochem. Photobiol.*, **452,** 801–810.

OPELZ G AND HENDERSON R for the Collaborative Transplant Study. (1993). Incidence of non-Hodgkin lymphoma in kidney and heart transplant recipients. *Lancet*, **342,** 1514–1516.

PARKIN DM, MUIR CS, WHELAN SL, GAO Y-T, FERLAY J AND POWELL J. (eds). (1992). *Cancer Incidence in Five Continents.* Vol. VI. IARC Scientific Publications No. 120. International Agency for Research on Cancer: Lyon.

PEARCE N AND BETHWAITE P. (1992). Increasing incidence of non-Hodgkin's lymphoma: occupational and environmental factors. *Cancer Res.*, **52,** (Suppl), 5496s–5500s.

RABKIN CS, BIGGAR RJ AND HORM JW. (1991). Increasing incidence of cancers associated with human immunodeficiency virus epidemic. *Int. J. Cancer*, **47,** 692–696.

RIVAS JM AND ULLRICH SE. (1994). The role of IL-4, IL-10, and TNFa in the immune suppression induced by ultraviolet radiation. *J. Leuk. Biol.*, **56,** 769–775.

SCHOTTENFELD D. (1982). Multiple primary cancers. In *Cancer Epidemiology and Prevention*, 1st ed., DR Schottenfeld and JR Fraumeni (eds) pp. 1025–1035. WB Saunders: Philadelphia.

SWERDLOW A AND DOS SANTOS SILVA I. (1994). *Atlas of Cancer Incidence in England and Wales 1968–85.* Oxford University Press: Oxford.

VAN DER ESCH EP, MUIR CS, NECTOUX G, MACFARLANE G, MAISONNEUVE P, BHARUCHA H, BRIGGS J, COOKE RA, DEMPSTER AG, ESSEX WB *et al.* (1991). Temporal change in diagnostic criteria as a cause of the increase of malignant melanoma over time is unlikely. *Int. J. Cancer*, **47,** 483–490.

VERMEER M, SCHMIEDER GJ, YOSHIKAWA T, VAN DEN BERG JW, METZMAN MS, TAYLOR JR AND STREILEIN JW. (1991). Effects of ultraviolet B light on cutaneous immune responses of humans with deeply pigmented skin. *J. Invest. Dermatol.*, **97,** 729–734.

YOSHIKAWA T, RAE V, BRUINS-SLOT W, VAN DEN BERG JW, TAYLOR JR AND STREILEIN JW. (1990). Susceptibility to effects of UVB radiation on induction of contact hypersensitivity as a risk factor for skin cancer in man. *J. Invest. Dermatol.*, **95,** 530–536.